# 100. Differences in influenza vaccination by gender identity and state-level gender equity policies: Data from the Behavioral Risk Factor Surveillance System, 2015-2019

**DOI:** 10.1093/ofid/ofac492.178

**Published:** 2022-12-15

**Authors:** Sarah N Cox, Mark A Fajans, Collrane J Frivold, Alyson J Littman, Jennifer E Balkus

**Affiliations:** University of Washington, Seattle, Washington; University of Washington, Seattle, Washington; University of Washington, Seattle, Washington; University of Washington, Seattle, Washington; University of Washington, Seattle, Washington

## Abstract

**Background:**

It is estimated that 1.4 million people identify as transgender and over 700,000 people identify as non-binary in the United States, many of whom face significant health disparities impacting health care access. Although previous studies have reported greater vaccine uptake in women compared to men, national-level estimates of influenza vaccine uptake among transgender and non-binary people are unknown. This study aims to characterize differences in influenza vaccination by gender identity and examine associations between vaccination status and state-based gender equality policies.

**Methods:**

We used cross-sectional data from adults aged 18 and older who participated in the 2015-2019 United States Behavioral Risk Factors Surveillance System surveys. Weighted prevalence differences (PDs) and associated confidence intervals (CIs) of being unvaccinated against influenza by self-reported gender identity were estimated using generalized linear regression models. We identified state policies on gender identity (Figure 1) as a potential effect modifier a priori and assessed through stratification.

**Figure 1.**
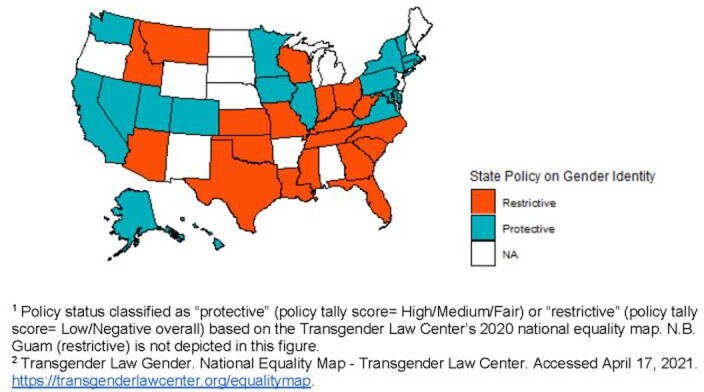
U.S. states implementing the SOGI module in Behavioral Risk Factors Surveillance System between 2015-2019, classified as having restrictive versus protective policies on gender equality (N = 38)

**Results:**

A total of 1,016,012 individuals met the inclusion criteria. Compared to cisgender women (unvaccinated prevalence=57.3%), the prevalence of being unvaccinated was significantly higher among cisgender men (64.4%; PD=7.0 per 100, 95% CI: 6.7-7.4), transgender women (65.4%; PD=8.1 per 100, 95% CI: 4.0-12.2), transgender men (64.6%; PD=7.3 per 100, 95% CI: 2.7-11.8), and non-binary individuals (64.6%; PD=7.2 per 100, 95% CI: 1.3-13.2). This pattern was similar among individuals living in protective states with more favorable gender-related policies compared to restrictive states with less favorable policies (Figure 2).

**Figure 2.**
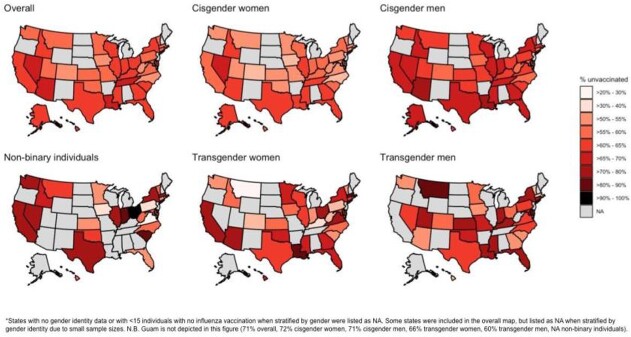
Weighted prevalence of U.S. adults unvaccinated against influenza by gender identity, Behavioral Risk Factors Surveillance System, 2015-2019

**Conclusion:**

Our results provide evidence of a disparity in influenza vaccine uptake by gender identity. To improve vaccine uptake among transgender and non-binary individuals, future research should focus on identifying barriers to and facilitators of vaccination by gender identity. These findings could be used to inform policies and public health interventions to improve vaccine uptake co-designed and implemented in partnership with the transgender and non-binary communities.

**Disclosures:**

**All Authors**: No reported disclosures.

